# Comparing Programming Sessions of Vim-DBS

**DOI:** 10.3389/fneur.2020.00987

**Published:** 2020-09-03

**Authors:** Sarah C. Reitz, Sebastian Luger, Sriramya Lapa, Michael Eibach, Natalie Filmann, Volker Seifert, Lutz Weise, Johannes C. Klein, Jun-Suk Kang, Simon Baudrexel, Johanna Quick-Weller

**Affiliations:** ^1^Department of Neurology, University Hospital, Frankfurt, Germany; ^2^Department of Neurosurgery, University Hospital, Frankfurt, Germany; ^3^Division of Neurosurgery, Dalhouse University Halifax, Halifax, NS, Canada; ^4^Institute of Biostatistics and Mathematical Modeling, University Hospital, Goethe University, Frankfurt, Germany; ^5^Nuffield Department of Clinical Neurosciences, University of Oxford, Oxford, United Kingdom

**Keywords:** DBS, essential tremor, ventralis intermedius nucleus, VIM, brain shift

## Abstract

**Background:** Essential Tremor (ET) is a progressive neurological disorder characterized by postural and kinetic tremor most commonly affecting the hands and arms. Medically intractable ET can be treated by deep brain stimulation (DBS) of the ventral intermediate nucleus of thalamus (VIM). We investigated whether the location of the effective contact (most tremor suppression with at least side effects) in VIM-DBS for ET changes over time, indicating a distinct mechanism of loss of efficacy that goes beyond progression of tremor severity, or a mere reduction of DBS efficacy.

**Methods:** We performed programming sessions in 10 patients who underwent bilateral vim-DBS surgery between 2009 and 2017 at our department. In addition to the intraoperative (T1) and first clinical programming session (T2) a third programming session (T3) was performed to assess the effect- and side effect threshold (minimum voltage at which a tremor suppression or side effects occurred). Additionally, we compared the choice of the effective contact between T1 and T2 which might be affected by a surgical induced “brain shift.”

**Discussion:** Over a time span of about 4 years VIM-DBS in ET showed continuous efficacy in tremor suppression during stim-ON compared to stim-OFF. Compared to immediate postoperative programming sessions in ET-patients with DBS, long-term evaluation showed no relevant change in the choice of contact with respect to side effects and efficacy. In the majority of the cases the active contact at T2 did not correspond to the most effective intraoperative stimulation site T1, which might be explained by a brain-shift due to cerebral spinal fluid loss after neurosurgical procedure.

## Introduction

Essential Tremor (ET) is a progressive neurological disorder characterized by postural and kinetic tremor most commonly affecting the hands and arms, sometimes the head and neck, and rarely the lower limbs (1). Its disease progression is highly variable (2), and there is some evidence of neurodegeneration with ET (3, 4). Medical treatment options for ET are limited (5). Up to 50% of patients do not respond to commonly used drugs. Moreover, side-effects are often limiting therapy.

Medically intractable ET can be treated by deep brain stimulation (DBS) of the ventral intermediate nucleus of thalamus (VIM) (6, 7), which is a safe and effective treatment modality (8–10). So far, there is only limited data about long-term efficacy of VIM DBS. A recent study showed that tremor severity worsens but also efficacy of DBS diminishes over time (11). Habituation to stimulation can also occur (12), indicative for this for example an increase of the total electric energy delivered (TEED) by the internal generator is observed (13). In contrast, other studies (the European long-term study) described long-lasting effects at 6 years (10, 14).

In surgery, the location with maximum tremor suppression and minimal side effects is sought (hereinafter referred to as “the effective contact”). Following Iandmark-guided stereotactic planning, intraoperative clinical testing confirms the effective contact (6).

Given worsening of tremor severity and reduction of DBS efficacy over time in VIM DBS, the question arises whether in addition to disease progression and habituation a change in the location of the effective contact plays a role. This could be due to motor circuit remodeling driven by tremulous activity or by the DBS itself.

The aim of this study was to investigate whether the effective contact location remains unchanged after surgery since we are more likely to expect a disease progression being responsible for tremor worsening.

## Methods

A total of 12 patients with severe, medically, and drug-refractory ET who met the diagnostic criteria of ET according to the consensus statement of the Movement Disorder Society (15) underwent VIM-DBS surgery between 2009 and 2017. Bilateral DBS electrodes were implanted into the VIM using landmark-guided stereotactic planning and intraoperative test stimulation (6). None of the patients included had electrode dislocation, hardware failure, or other neurological disease. Furthermore, any tremor-specific medications were withdrawn before testing. Ten of these patients were available for follow-up (one lost to follow-up, one died).

### Surgical Procedure

The leksell G- frame was used for all surgical procedure. The frame was mounted to the head with four pins after local anesthesia was subcutaneously applied (mecaine 2%). Two pins were placed frontal and two occipital. After the frame was mounted, a CT scan was performed. MRI and CT scan was fused, the planning procedure had already taken place several days before. Standard VIM coordinates were used as target point, they were slightly altered for the individual patient to avoid complications such as hemorrhage. In all cases the target point was frontal. After adjusting the frame to the calculated coordinates according to the stereotactic plan, skin incision was performed by the surgeon.

All burrholes were placed frontal after a 3 cm skin incision. Burrholes were ~1 cm in diameter. A burrhole cover (Medtronic) was used in all cases. The microdrive was attached to the instrument holder of the frame and the electrode was placed for testing. Finally, after testing, the electrode was fixed to the burrhole cover. The same procedure took place on the other side of the patients head. In all cases the first implantation was performed on the clinical worse side. After skin closure the frame was detached from the patients head. General Anesthesia was applied for IPG (Impulse Generator).

Implantation Intraoperative definition of the effective contact (herein after referred to the intraoperative programming session, T1) was made by evaluating efficacy and side effects at different levels of the five trajectories. The DBS electrode was implanted at the effective target point and connected to the DBS system [Medtronic^©^ 3387 electrodes (4 contacts, contact pitch 3 mm) connected to Kinetra or Activa PC neurostimulators (Medtronic^©^, Minneapolis, MN)]. Sixty percentage of all electrodes (12/20) were implanted into the central trajectory, another 35% (7/20) into the medial and 5% (1/20) into the anterior trajectory.

To compare the electrode position at T1 with the following programming sessions T2 and T3 the contacts designation in relation to the inserted depth was determined based on the information given from the Medtronic^©^ implant manual (https://www.medtronic.com/us-en/healthcare-professionals/products/neurological/deep-brain-stimulation-systems/activa-rc.html) and by means of the intraoperative testing protocol.

The first post-operative programming session (T2) for establishing stimulation parameters took place between 4 and 6 days after surgery when the microlesion effect of the implantation procedure had faded. The algorithm has been described previously by Volkmann et al. for patients receiving DBS in Parkinson's disease (16). However, stimulation parameters were adapted for VIM (pulse width 60 μs, stimulation frequency 130 Hz). For every contact the effect threshold (minimum voltage at which a tremor suppression occurred) and the side effect threshold (minimum voltage at which a side effect occurred). For all programming sessions patients did not take tremor medication for at least 12 h.

Patients were clinically evaluated at follow-up and a third programming session (T3) was performed. For the examination at T3 DBS had been switched off for >12 h.

At T3 patients were also assessed with the Fahn-Tolosa-Marin Tremor Rating Scale (FTMRS) to assess tremor severity (overall score 144, higher values indicate more impairment. Part A categorizing tremor modalities and locations, maximum score 28; part B including handwriting, pouring, and drawing the Archimedes spiral, maximum score 36; part C reflects quality of life, maximum score 28) (17). Evaluation of FTMRS was made in OFF and ON stimulation condition.

**Figure 1:**
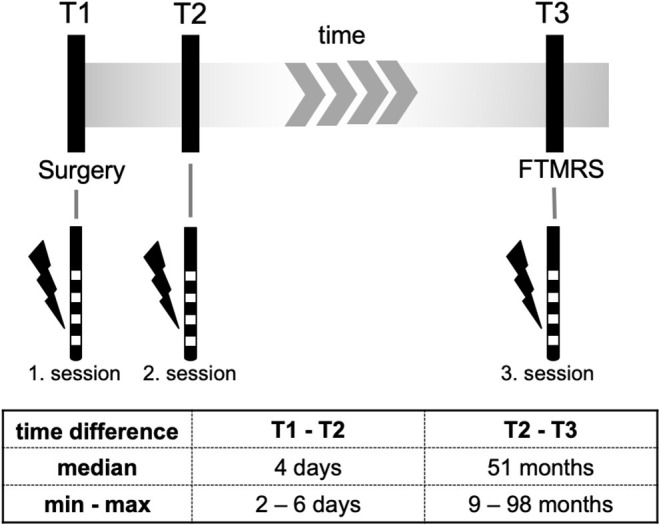
Study design.

All clinical tests at T3 were done by trained physicians (S.R., S.L.) and supervised by a senior movement disorder specialist (J-S.K.). Data from intraoperative contact testing T1 as well as from T2 were processed retrospectively based on available data. Figure 1 gives an overview of the study design.

Estimation of the total electric energy delivered (TEED) was calculated for T2 and T3 according to the following formula [(voltage^2^ × pulse width × frequency)/impedance] × 1 s (18). The comparison was made using exact Wilcoxon Rank tests. Because of the small number of cases exact McNemar tests were used to compare differences in the choice of the effective contact. Comparing the occurrences threshold of tremor suppression and undesired side effects exact Wilcoxon Rank tests were performed. Mean values are given in mean ± standard deviation (SD). The interquartile range (IQR) is given for medians. All tests were two-sided and the level of statistical significance was set at *p* ≤ 0.05. Statistical analyses were conducted with SPSS (version 26, IBM, Armonk, NY).

The study was approved by the local ethics committee and conducted in accordance with the Declaration of Helsinki. Written informed consent to participate in this assessment was given by all patients.

## Results

Ten consecutive patients with ET (male/female *n* = 7/3) who received bilateral stimulation of the VIM were included in this study. Every patient was treated with at least one tremor specific drug prior to surgery. The mean number of drugs before surgery was 3.4 ± 0.4. Four out of ten patients (4/10) activate the stimulation only temporarily (hourly intervals) due to side effects. In 8 patients a speech therapist objectified dysarthria and/or dysphagia as unwanted side effects. Other side effects were gait disorder and intermittent dyskinesia. There was no difference of TEED between T2 and T3 (*z* = 0.663, *p* = 0.557). Clinical data are shown in Table 1. One patient's T2-session-data was missing.

**Table 1:** Clinical data and demographics, *n* = 10.

	**Mean ± SD**	**Median (IQR)**
Female/male	3/7	
Age at clinical onset	38 ± 24.58	50 (10–60)
Age at first visit, [y]	61.3 ± 7.44	63 (59.25–66)
Age at surgery (T1) [y]	61.8 ± 5.79	63 (59.25–75)
Age at T3 [y]	67.8 ± 8.69	67.5 (63.25–74.75)
Duration of tremor at T3 [y]	25.1 ± 21.20	18.5 (6–41)
Time between T1 and T2 [d]	3.8 ± 1.75	4 (2–5.25)
Time between T2 and T3 [mo]	49.7 ± 30.04	51 (23.75–75.5)
TEED at T2	76.5 ± 46.74	66.1 (45.9–121.48)
TEED at T3	94.9 ± 96.02	59.7 (45.53–115.42)
Number of drugs at T3	0.6 ± 0.70	0.5 (0–1)
Number of drugs (before surgery)	3.4 ± 1.35	3.5 (2.75–4)
Positive family history unkown/yes/no	2/3/5	
Alkohol responsitivity unkown/yes/no	3/5/2	
With/without medication at T3	5/5	
Rarely or not at all stim-on (because of side effects)	4	

*Data is given in mean and standard deviation (SD) as well as median and inter quartile range (IQR). Time points T1, T2, T3 correspond to the programming sessions*.

**Figure 2:**
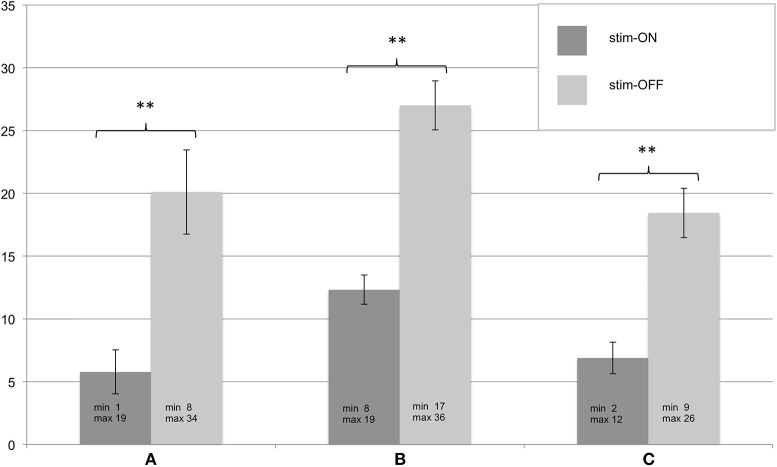
**(A–C)** Assessment of the Fahn-Tolosa-Marin Tremor Scale (FTMRS) at T3. FTMRS was tested in stim-ON and stim-OFF condition, standard errors of the mean are depicted as error bars, significant differences of the exact Wilcoxon Rank tests are shown with ** (*p* < 0.01). Additionally minimum and maximum of the evaluated FTMRS score are given.

All patients in whom the FTMRS could be determined at T3 (*n* = 9) showed significant improvement in tremor severity in all tested areas (A, B, C) during stim-ON compared to stim-OFF (see Figure 2). In one patient the assessment was not successful due to a lack of cooperation.

Comparing the effect for first occurrence of tremor suppression between T2 and T3 (“effect threshold”) revealed no significant differences for 7 out of 8 tested contacts, for one contact a trend was revealed. Threshold comparison of unwanted side effects showed a significant difference for one contact, for 7 out of 8 tested contacts no significant difference was revealed. Data is shown in Table 2.

**Table 2:** Comparison of the occurrences threshold of tremor suppression and undesired side effects (exact Wilcoxon Rank tests).

	**Contact**	**Effect threshold**	**Side-effect threshold**
VIM left	0	*z* = −1.414, *p* = 0.312	*z* = −632, *p* = 0.656
	1	*z* = −1.069, *p* = 0.5	*z* = −1.5779, *p* = 0.156
	2	*z* = −0.962, *p* = 0.5	*z* = −2.184, *p* = 0.031
	3	*z* = −0.742, *p* = 0.563	*z* = −1.194, *p* = 0.250
VIM right	8	*z* = −2.236, *p* = 0.063	*z* = −0.106, *p* = 1
	9	*z* = −1.414, *p* = 0.312	*z* = −0.853, *p* = 0.484
	10	*z* = −1.225, *p* = 0.25	*z* = −1.703, *p* = 0.125
	11	*z* = −1.511, *p* = 0.25	*z* = 0.0, *p* = 1

In the majority of the cases the active contact at T2 did not correspond to the most effective intraoperative stimulation site T1 (VIM left: change/no change 6/3, VIM right: change/no change 7/2). A cumulative analysis of all active contacts of the VIM stimulation of each hemisphere together suggested an overall change in the choice of the effective pole between the intraoperative testing T1 and the first clinical programming session T2 (change/no change 13/5). Here, the McNemar test could not be applied due to dependency of cumulated data. However, the exact McNemar test for the VIM of each hemisphere showed only a trend (*p* = 0.125). Looking at the change in direction along the electrode (cranial vs. caudal) when choosing the effective contact in the comparison between T1 and T2, the direction has never changed more than 2 contacts (0.83 ± 0.618). Regarding all changed contacts (13 in total), tthere is a trend toward the cranial direction (11/13, 84.6%). Data is shown in Table 3.

**Table 3:** Absolute change of the effective contact comparing the different programming sessions as well as change in direction of the effective contact comparing T1 and T2.

			**T1–T2**						**T2–T3**	**Exact McNemar test**
			**Change abs**		**Change cranial (+)**		**Change caudal (–)**		**Change abs**.	
			***n***	**Mean ± SD**	***n***	**Mean ± SD**	***n***	**Mean**	***n***	
*n =* 9	Left	No change	3	–	–	–	–	–	7	*p* = 0.125
		Change	6	0.78 ± 0.667	5	1.20 ± 0.400	1	1	2	
*n =* 9	Right	No change	2	–	–	–	–	–	6	*p* = 0.125
		Change	7	0.89 ± 0.601	6	1.17 ± 0.373	1	1	3	
*n =* 2*9	Cum.	No change	5	–	–	–	–	–	13	(*)
		Change	13	0.83 ± 0.618	11	1.18 ± 0.405	2	1	5	

(*) Here, the McNemar test could not be applied due to dependency of cumulated data.

## Discussion

In this retrospective analysis we investigate whether the location of the effective contact in VIM-DBS for ET changes over time, indicating a mechanism of loss of efficacy that goes beyond progression of tremor severity, or a mere reduction of DBS efficacy.

Nevertheless, in the literature reduction of DBS benefit in ET has been reported previously (11, 19–21), but the mechanism remains unclear. Cury et al. assumed a VIM stimulation benefit in ET up to 18 years, although reduced efficacy was observed 10 years after surgery (22). As a potential mechanism, habituation to DBS (4, 6, 9, 23, 24), as well as an ongoing and progressive neurodegenerative disease (3, 4, 25) or a combination of both have been suggested.

An important aspect to interpret our results is how efficacy is defined. One way of defining, which has been used before, is to compare the tremor severity under both conditions (stim-ON and stim-OFF) over the long term (11). In this case, a deterioration in efficacy can also be masked by the neurodegenerative, respectively, disease progression aspect. For our data an evaluation based on this definition was unfortunately not possible due to the lack of baseline FTMRS and thus the evaluation of the tremor over time.

However, if efficacy is defined by the fact that side effects and effect thresholds in the programming sessions for every single contact remain the same over time, we would assume that DBS efficacy remains the same, too. In our sample, the comparison of FTMRS under stim-ON and stim-OFF conditions showed a relevant improvement of symptoms even after a median of 4.25 years, meaning the patients continued to benefit from the DBS. As an indirect evaluation benchmark, no significant difference between T2 and T3 in the comparison of the TEED was observed, which does not suggest increased energy consumption. Furthermore, regarding the threshold for occurrence of side effects in 7 out of 8 tested contacts no significant difference (only for one contact a trend) over time was observed. Regarding the threshold for effects on tremor severity no significant difference was observed. An observed difference in the threshold of one contact under both conditions each has to be considered an outlier. The small sample results in a cautious statement of the just mentioned. Unfortunately, no randomized long-term studies comparing ET patients with DBS vs. non-DBS exists.

According to current consensus our conclusion leads us to the assumption, that at least in some patients the observed tremor worsening might be caused by disease progression rather than tolerance of chronic stimulation (22, 26, 27).

Because the effect of DBS depends on accurate electrode positioning, suboptimal positioning of the DBS electrodes may account for loss of benefit in patients who initially respond well to the treatment (21). This phenomenon manifests in the first 6 months of DBS-therapy. However, in contrast to this study, we were able to show that the choice of effective contact has not changed over a long period of time (T2 vs. T3, median 51 months). The change in choice of the effective contact comparing intraoperative stimulation with the first clinical programming session could be explained by a brain shift that took place while surgery, mediated by loss of cerebrospinal fluid when opening the dura. This observed complication was first mentioned by Gerdes et al. (28) as “brain sinking” in the stereotactic procedure (28). Although there are some studies on brain shift [e.g., (29–31)] the impact of this phenomenon on DBS is not well-studied. A minor finding was a high incidence of dysarthria or dysphagia as side effect. Although usually mild, this warrants regular swallow assessment (32).

Limitations of this exploratory study include the small sample size, and its retrospective design. Tremor scale assessments were performed by two different examiners, however FTMRS has been demonstrated to maintain good interrater reliability (33). For future studies, a blinded assessment (for example as video-based rating) of tremor severity can be considered.

Compared to immediate postoperative programming sessions in ET-patients with VIM-DBS, long-term evaluation showed no relevant change in the choice of contact with regard to side effects and efficacy. VIM-DBS showed continuous efficacy in tremor suppression during stim-ON compared to stim-OFF condition.

A brain shift after surgery can be discussed as possible explanation for an overall change in the choice of the effective pole between the intraoperative testing T1 and the first clinical programming session T2, although this does not appear to affect efficacy of DBS. With a better understanding of ET disease progression, we may also be able to understand the effectiveness of interventional therapies and thus improve them.

## Data Availability Statement

The raw data supporting the conclusions of this article will be made available by the authors, without undue reservation.

## Ethics Statement

The studies involving human participants were reviewed and approved by Ethics committee Goethe University Hospital Frankfurt. The patients/participants provided their written informed consent to participate in this study.

## Author Contributions

JQ-W and J-SK conceived the presented idea and developed the experimental design together with SR. SR performed Programming session T3 and prepared the submitted manuscript. Clinical examinations via FTMRS were performed by SR and SLu. NF was involved in the statistical analysis of the data. All authors listed have made a substantial, direct and intellectual contribution to the work, and approved it for publication. All authors contributed to the article and approved the submitted version.

## Conflict of Interest

The authors declare that the research was conducted in the absence of any commercial or financial relationships that could be construed as a potential conflict of interest.

## Abbreviations

DBS: deep brain stimulation
ET: Essential Tremor
SD: standard deviation
TEED: total electrical energy delivered
VIM: ventral intermediate nucleus of thalamus.
